# Clinical features and severe outcome predictors of COVID-19 vaccine breakthrough infection among hospitalized patients: results from Egypt severe acute respiratory infections sentinel surveillance, 2021–2022

**DOI:** 10.1186/s12879-023-08097-z

**Published:** 2023-03-06

**Authors:** Amr Kandeel, Manal Fahim, Ola Deghedy, Walaa Alim, Mohammad Abdel Fattah, Salma Afifi, Amira Mohsen, Khaled Abdelghaffar

**Affiliations:** 1 Ministry of Health and Population, Cairo, Egypt; 2 Ministry of Health and Population Consultant, Cairo, Egypt; 3 National Research Center, Cairo, Egypt

**Keywords:** Severe acute respiratory syndrome virus, Vaccines, Hospitalizations, Breakthrough infections, Egypt

## Abstract

**Background:**

COVID-19 vaccines are effective against infections and outcomes; however, breakthrough infections (VBT) are increasingly reported, possibly due to waning of vaccine-induced immunity or emerging variants. Most studies have focused on determining VBT rate based on antibody levels. This study aims at describing clinical features, risks, time trends, and outcomes of COVID-19 VBT among hospitalized patients in Egypt.

**Methods:**

Data of SARS-CoV-2 confirmed patients hospitalized in 16 hospitals was obtained from the severe acute respiratory infections surveillance database, September 2021-April 2022. Data includes patients’ demographics, clinical picture, and outcomes. Descriptive analysis was performed and patients with VBT were compared to not fully vaccinated (UPV). Bivariate and multivariate analyses were performed using Epi Info7 with a significance level < 0.05 to identify VBT risk factors.

**Results:**

Overall, 1,297 patients enrolled, their mean age 56.7 ± 17.0 years, 41.5% were males, 64.7% received inactivated, 25.% viral vector, and 7.7% mRNA vaccine. VBT was identified in 156(12.0%) patients with an increasing trend over time. VBT significantly was higher in (16–35 years) age, males, in those who received inactivated vaccine compared to corresponding groups of UPV (14.1 vs. 9.0%, p < 0.05 and 57.1 vs. 39.4%, p < 0.001 and 64.7 vs. 45.1, p < 0.01 respectively). Whereas receiving mRNA vaccine was significantly protective against VBT (7.7 vs. 21.6%, p < 001). VBT patients tend to have shorter hospital stays and lower case fatality (mean hospital days = 6.6 ± 5.5 vs. 7.9 ± 5.9, p < 0.01 and CFR = 28.2 vs. 33.1, p < 0.01 respectively). MVA identified younger ages, male gender, and inactivated vaccines as risks for VBT.

**Conclusion:**

The study indicated that COVID-19 vaccines significantly reduce hospital days and fatality. VBT trend is on the rise and males, young ages, and inactivated vaccine receivers are at higher risk. Caution regarding relaxation of personal preventive measures in areas with higher or increasing incidences of COVID-19, particularly for the at-risk group even if they are vaccinated. The vaccination strategy should be revised to reduce VBT rate and increase vaccine effectiveness.

## Introduction

The ongoing COVID-19 pandemic evolved in 2019 ranks among the world’s deadliest epidemics. Officially between January 2020 and December 2021 SARS-CoV-2 has claimed 5.94 million lives, yet credible estimates place the pandemic’s true death toll closer at 18.2 million [1]. Amid the coronavirus crisis, safe and effective vaccines offer the most promising way out and are a game-changing tool for ending the COVID-19 pandemic. It is hugely encouraging to see so many vaccines proving and going into development.

While people who are fully vaccinated against COVID-19 have a significantly reduced risk of severe illness and fatality, some hospitalizations and deaths have been reported among fully vaccinated people. Aware of the magnitude of the current public health emergency and of the importance of facilitating the availability of vaccines to prevent COVID-19, and that the public will trust, FDA has granted Emergency Use Authorization (EUA) for different COVID-19 Vaccines. COVID-19 vaccines were evaluated through many clinical trials conducted according to the rigorous FDA standards in tens of thousands of study participants to determine its safety and effectiveness [2]. Multiphase randomized controlled trials (RCT) have shown high vaccine efficacy against symptomatic COVID-19 infection. However, RCT is usually performed under optimal conditions where vaccine storage and delivery are monitored, and participants usually in good health or selected for a specific health status [3].

Recently, SARS-CoV-2 vaccine breakthrough (VBT) infections have increasingly been reported in fully vaccinated patients. However, the fully vaccinated individuals remained less likely to become hospitalized and the number of fully vaccinated cases with breakthrough SARS-CoV-2 infection hospitalization was significantly lower when compared to the number of unvaccinated cases [4]. Observational studies are needed to understand the impact of waning immunity, viral variants, and other determinants of changing vaccine effectiveness against COVID-19 severity in the real-world conditions [5]. In addition, data is needed to characterize the clinical characteristics of patients hospitalized with COVID-19 after vaccination and monitor the performance of vaccines in preventing disease severity and outcome [6]. Monitoring vaccine effectiveness is necessary to determine how long restrictions will be required and decide on the need for vaccine booster doses or changes to the vaccine formulations or policy.

Globally, mass vaccination campaigns are underway to combat the COVID-19 pandemic with approximately 60% of world population being fully vaccinated [7]. In Egypt, vaccination was initiated in May 2021, with vaccination coverage rate reached 44.7% as of May 2022. Two inactivated vaccines (two doses: Sinovac and Sinopharm) and three viral vector vaccines (AstraZeneca, Sputnik V (two doses) and Johnson& Johnson (one dose)) and two mRNA vaccines (Pfizer and Moderna (Two doses)) are used. The most widely available and used vaccines in Egypt are the inactivated vaccines, followed by viral vector vaccines and mRNA vaccines (Ministry of Health and Population personal communication).

This study aims to evaluate the effectiveness of different COVID-19 vaccines in preventing severe outcomes and identify factors contributing to breakthrough infection after full vaccination among hospitalized patients with SARS-CoV-2 using the severe acute respiratory infections (SARI) sentinel surveillance data.

## Methods

### Study sites and subjects

SARI was established in Egypt in 2007 to identify viral causes of acute respiratory diseases. Currently, the system is operating in a wide network of 16 governmental hospitals and regional laboratories covering ten governorates within all Egyptian regions. As a result of the emergence of the COVID-19 pandemic in 2019, the system was adapted to include SARS-CoV-2 in the laboratory testing panel to address the emerging challenges.

WHO case definition is used to identify and enroll SARI patients after consenting to participation. During the pandemic, surveillance teams were instructed to enroll every fifth admitted patient with SARI symptoms to adapt to the increased number of patients with acute respiratory infections (ARI) symptoms.

### Data collection

Enrolled patients are interviewed using a standard data collection tool that includes patients’ demographics, clinical picture, ARI risk factors, disease severity, and outcome. The tool was updated to include information on vaccination status and vaccine types following the introduction of COVID-19 vaccines in Egypt. Vaccination data are collected either from vaccination cards or as stated by patients if no cards are available at the time of the interview. An online application was developed for COVID-19 data entry and reporting, data is merged into a common ARI database at the central level.

Data of all hospitalized patients confirmed for SARS-CoV-2 by RT-PCR from September 2021-April 2022 was extracted from the surveillance database.

### Laboratory procedures

Nasopharyngeal/oropharyngeal swabs are collected and shipped weekly to Central Public Health Laboratories. RT-PCR are used to confirm SARS-CoV-2, influenza types and subtypes, and Respiratory syncytial Virus (RSV). To avoid the delay in identifying and treating COVID-19 cases, two NP/OP swabs are collected from each patient, one immediately tested for COVID-19, and the other kept in nitrogen tank and transferred to the Central Public Health Laboratories (CPHL) on a weekly basis for routine influenza and RSV testing.

### Case definitions

Patients who received at least one dose of the COVID-19 vaccine were classified as “vaccinated”, and those who did not complete the vaccination schedule were classified as “partially vaccinated”. Patients were categorized as “fully vaccinated” if they have received the required dose(s) of a COVID-19 vaccine at least 14 days from the single-dose vaccine or the second dose in the two-doses vaccines. The partially or unvaccinated patients were categorized as “not fully vaccinated” group. Patients who acquire SARS-CoV-2 infection after being fully vaccinated are termed “patients with VBT”.

### Data analysis

Descriptive data analysis with frequencies and rates was performed for all COVID-19 lab-confirmed hospital-admitted patients at the sentinel sites. In order to identify risk factors for breakthrough infection, bivariate analysis was used to compare demographics, comorbidity, and vaccine type between patients with VBT and those without VBT. Binary logistic regression with a significance level < 0.05 was performed in order to exclude the confounders. The “fully vaccinated” patients were compared to the “not fully vaccinated” regarding disease severity and outcome in terms of ICU admission, the need for ventilation, and death at the hospital to assess different vaccines’ effectiveness in preventing severe disease and mortality. Pearson`s chi-square test and t-student test with a significance level < 0.05 were used to test the association. Analysis was conducted using epi info-7 software.

## Results

Between September 2021 and April 2022, a total of 3,414 patients with SARI symptoms were enrolled including 1,447 (42.4%) positive for SARS-CoV-2 by RT-PCR. Of the SARS-CoV-2 confirmed patients, 1,297 (89.6%) had completed vaccination data (Table 1).

**Table 1:** Characteristics of study subjects

Characteristic		Number of patients	Percent
Total admitted		3414	NA
Total admitted with confirmed COVID-19		1,447	42.4
COVID-19 patients with complete vaccination data		1,297	89.6
Vaccinated patients		207	16.0
No. of doses			
	one dose	45	21.7
	two doses	158	76.3
	three doses	4	1.9
Vaccine breakthrough infection		159	12.3
By vaccine type			
	Inactivated	101	63.5
	Viral vector	42	26.5
	mRNA	12	7.5
	Type unknown	4	2.5
By time after vaccination			
	Within one month	12	7.5
	1-<2 months after	25	15.7
	2-<3 months after	34	21.4
	3-<4 months	18	11.3
	4-<5 months after	21	13.2
	5-<6 months after	13	8.2
	> 6months after	19	11.9
	Unknown	17	10.7

Their mean age was 56.7 ± 17.0 years, with 68.9% > 50 years, 41.5% were males, and 47.3% are having comorbidity (Table 2).

**Table 2:** Demographic characteristics of COVID-19 hospitalized patients by vaccination status

Characteristics		All patients (n = 1297)		Vaccine breakthrough (n = 159)		No breakthrough (n = 1138)			OR	95% CI	P value
		No.	Percent	No.	Percent	No.		Percent			
Age group											
	< 5	22	1.7	0	0.0	22	1.9		NA	NA	NA
	5–15	3	0.2	1	0.6	2	0.2		NA	NA	NA
	16–35	125	9.6	23	14.5	102	9.0		1.717	1.06–2.79	0.018
	36–50	253	19.5	25	15.7	228	20.0		0.744	0.47–1.17	0.099
	51–65	493	38.0	58	36.5	435	38.2		0.928	0.66–1.31	0.337
	> 65	401	30.9	52	32.7	349	30.7		1.099	0.77–1.57	0.300
Gender											
	Males	538	41.5	91	57.2	447	39.3		2.069	1.48–2.89	< 0.001
	Females	759	58.5	68	42.8	691	60.7				
-Co-morbidity											
	Diabetes	437	33.7	70	44.0	367	32.2		1.652	1.18–2.31	0.002
	Cardio-vascular	319	24.6	54	34.0	265	23.3		1.694	1.19–2.41	0.002
	Pulmonary	64	4.9	12	7.5	52	4.6		1.705	0.89–3.27	0.061
	Other*	53	4.1	8	5.0	45	4.0		1.287	0.60–2.78	0.256
Vaccine type (n = 202)											
	Inactivated	124	61.4	101	65.2	23	48.9		1.952	1.01–3.78	0.025
	Viral vector	55	27.2	42	27.1	13	27.7		0.972	0.47–2.02	0.465
	mRNA	23	11.4	12	7.7	11	23.4		0.275	0.11–0.67	0.003

*Including kidney and liver diseases.

Of the 1,297 confirmed COVID-19 hospital admitted patients, 207 (16.0%) had received at least one dose of COVID-19 vaccine. Fully vaccinated represent 12.3% (159/1,297) of all hospitalized patients with COVID-19 and fatality among them represent 8.0% (25/312) of the in-hospital deaths due to COVID-19.

Of 159 patients with COVID-19 breakthrough infection, 101 (63.5%) received inactivated vaccines, 42 (26.5%) viral vector, 12 (7.5%) mRNA vaccine type, while 4 (2.5%) were unable to mention or document the type of vaccine they received. More than half of patients with breakthrough infection had been infected within four months of receiving the full vaccine doses (Table 1).

Bivariate analyses showed that vaccine breakthrough infections were more common among younger patients (16–35 years old), males, those with diabetes, cardiovascular disease compared to corresponding groups of the “not fully vaccinated”. (14.5% vs. 9.0%, p < 0.05 and 57.2% vs. 39.3%, p < 0.001 and 44.0% vs. 32.2%, p < 0.01 and 34.0% vs. 23.3%, p < 0.01 and 65.2% vs. 48.9%, p < 0.05 respectively). Breakthrough infection rates were higher among those who received inactivated vaccines (65.2% vs. 48.9%, p < 0.05) and lower among those who received mRNA vaccines(7.7% vs. 23.4%, p < 01), while no significant difference was observed among those who received viral vector vaccines (Table 2). By multivariate analysis, only inactivated vaccine (OR, 95% CI = 96.4, 54.7-169.8), age group 15–35 years (OR, 95% CI = 3.4, 1.8–6.8), male gender (OR, 95% CI = 2.3,1.4–3.7), cardiovascular disease (OR, 95% CI = 2.2, 1.2–3.9) remained significant (Table 3).

**Table 3:** Factors associated with vaccine breakthrough by multivariate analysis

	OR	95% CI	P value
Inactivated vaccines	96.37	54.70-169.77	< 0.001
Age 16–35	3.44	1.75–6.76	< 0.001
Male gender	2.27	1.42–3.65	< 0.001
Cardiovascular disease	2.16	1.21–3.85	0.009

**Fig. 1:**
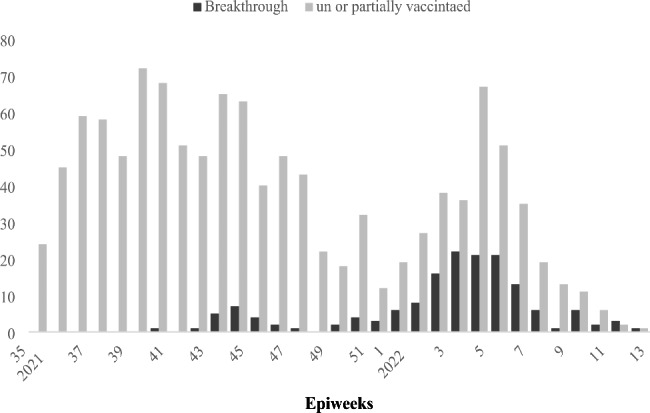
Distribution of VBT cases by time,Egypt,2021–2022

The number of vaccine breakthrough infections increases over time, peaking with the increase of COVID-19 infections (Fig. 1).

When evaluating the effectiveness of COVID-19 vaccine for the prevention of severe disease and death during hospitalization, it was found that fully vaccinated patients had significantly lower mean hospital days and case fatality rate than the “not fully vaccinated” (6.6 ± 5.5 vs. 7.9 ± 5.9, p < 0.01 and 15.7% vs. 25.2%, p < 0.01 respectively). Whereas no significant difference found between the two groups regarding rate of pneumonia infection, bilateral pneumonia, ICU admission, ICU days, mechanical ventilation, and ventilator days (Table 4).

**Table 4:** Clinical picture and COVID-19 disease course by vaccination status

Characteristics		All patients (n = 1297)		Fully vaccinated (n = 159)		Not fully vaccinated (n = 1138)		OR	95% CI	P value
		No.	Percent	No.	Percent	No.	Percent			
Pneumonia		979	75.5	119	74.8	860	75.6	0.96	0.66–1.41	0.42
	Unilateral	338	26.1	44	37.0	294	34.2	0.88	0.60–1.32	0.27
	Bilateral	641	49.4	75	63.0	566	65.8			
ICU admission		309	23.8	38	23.9	271	23.8	1.00	0.68–1.48	0.49
Mean ICU days ± SD		5.2 ± 5.3		4.6 ± 3.8		5.2 ± 5.4				0.08
Ventilated		106	8.2	9	5.7	97	8.5	0.64	0.32–1.30	0.11
Mean ventilation days ± SD		2.5 ± 3.4		3.0 ± 3.7		2.0 ± 2.9				0.08
Hospital days ± SD		7.7 ± 5.9		6.6 ± 5.5		7.9 ± 5.9				< 0.01
In-hospital death		312	24.1	25	15.7	287	25.2	0.55	0.35–0.87	< 0.01

## Discussion

Despite the very high level of COVID-19 vaccines efficacy and effectiveness, still, some hospitalizations and deaths due to COVID-19 have been reported among fully vaccinated people [8]. A growing risk of breakthrough infection has been documented in long-term follow-up studies of vaccine trial participants, along with a steady decline in antibody levels among vaccinated persons [9, 10]. Unlike most studies that measured antibody levels to determine vaccine breakthrough rates in vaccinated individuals, this study investigates the characteristics of hospitalized patients with COVID-19 breakthrough infections in real-life settings [3].

Although unvaccinated people continue to represent the vast majority of COVID-19 hospitalizations, yet the rate of breakthrough infections is increasingly reported [8]. Similarly, this study identified a rising number of breakthrough infections over time. Possible reasons could include increased numbers of vaccinated persons, waning of vaccine-induced antibody levels, emerging variants with diminished susceptibility to vaccine-induced antibodies, and reducing mitigation recommendations for the community [11].

The study found that the rate of breakthrough infections peaks with the increase in COVID-19 infections, similar to other studies which reported the highest breakthrough incidence rates coincident with high positive positivity rate in the general population [12]. This might be explained by the reduced vaccine-induced immunity during the emergence of new variants where fully vaccinated people remain susceptible to infection. Studies comparing the incidence of breakthrough infections between different variant dominance periods found higher incidence in the delta period than in the alpha period [13, 14]. This should raise concern about COVID-19 vaccines effectiveness as new SARS-CoV-2 variants emerge.

Compared to other studies that reported breakthrough rates ranging from 9 to 35%, this study reported a relatively low proportion of fully vaccinated patients among hospitalized patients with COVID-19 [12, 15, 16]. However, comparing rates of breakthrough remains challenging because of study-specific differences including characteristics of study population and methods, Country-specific vaccination campaigns, vaccines used, seasonal effects, protective measures, and vaccination coverage [12]. Monitoring the change in rates of breakthrough infections in one location using surveillance data may provide early insight into vaccine effectiveness that can be confirmed by controlled studies.

Our findings show that people hospitalized with COVID-19 who were fully vaccinated are more likely to be younger individuals. The age at which individuals are more likely to develop a breakthrough infection is controversial, many studies reported higher rates of breakthrough among elderly hospitalized patients [8, 17, 18]. However, in accordance with our results, others concluded that young people are more liable to breakthrough infections [12, 19]. It was suggested that the controversy between studies could be related to the type of vaccine used. Yi S et al. found higher breakthrough rates in younger recipients of AstraZeneca, Janssen and Moderna, while this rate was higher in elderly Pfizer-BioNTech recipients. Wearing masks indoors regardless of vaccination status and age is recommended for individuals living in communities where SARS-CoV-2 transmission rates are substantial or high. [20].

Studies reported no difference in incidence of breakthrough infection by gender [12], while others suggested male predominance in accordance with our study. The gender difference was explained by physiological, genetic factors or behavioral factors. Female protection against breakthrough infection was connected to the higher expression of ACE-2 receptors found in females as compared to males. These receptors get downregulated following the entry of virus particles into the cells, prevent further viruses from entering into the host cells and rendering females less vulnerable to severities of COVID-19 [17].

In accordance with other studies, our data found that patients with comorbidities especially those with diabetes and cardiovascular disease are more liable to have a breakthrough infection [21]. It is important for people with comorbidities to stick to recommended preventive measures against COVID-19 infection despite being vaccinated.

In this study we found that the incidence of breakthrough infections among vaccine recipients varied significantly according to the type of vaccine used. The same finding was reported by Stouten et al., who found that viral vector vaccines had a higher breakthrough rate than mRNA vaccines [12]. Our study also found that inactivated vaccines had the highest rate of breakthrough infections, which was not tested in previous studies. This could be related the higher percentage of inactivated vaccines received among Egyptian population. Vaccines formulation or policy or both should be updated based on these findings.

Thomas et al., recently reported a gradual decline in vaccine efficacy of 86 to 100% across countries and in populations with diverse ages, sexes, race, or ethnic groups [9]. In this study, we found that more than half of the breakthrough infections occurred within four months from date of full vaccination. This should raise concerns regarding losing efficacy of vaccines over time and suggest considering modify vaccine formulation to enhance vaccine efficacy and effectiveness.

The decline in vaccine efficacy over time and the continuous gradual waning in population vaccine-induced immunity could also explain the insignificant difference found between the fully and not fully vaccinated patients regarding disease severity found in this study. In addition, high viral loads found in fully vaccinated individuals when infected with SARS-CoV-2 [18]. However, earlier studies reported less severe disease course and fatality in the fully vaccinated individuals [4]. In accordance with these results, our study proved that vaccines are still effective in reducing hospital stay and fatality rates among COVID-19 patients. Recent reports from the United States indicated a growing proportion of COVID-19 deaths among vaccinated individuals, however vaccination remains the best option to lower risk of severe disease course, hospitalization, and death from COVID-19 [22]. Monitoring vaccine effectiveness in preventing COVID-19 severity and fatality among breakthrough infection is essential to update vaccination policy.

### Study limitations

This study results are subject to at least four limitations. First, data of hospitalized patients are insufficient to draw conclusions about the effectiveness of COVID-19 vaccines. Second, vaccinated persons are likely to represent a larger proportion of hospitalized COVID-19 cases as the rate of vaccination increases in population. Third, the demographics of cases likely reflect those of hospitalized patients rather than community, and fourth, a recall bias may occur since vaccination data were collected from the patient’s statement when vaccination cards were not available.

## Conclusion

We report an increase in breakthrough infections among hospitalized patients with COVID-19 in Egypt, which usually occur within four months of completing full vaccination. Breakthrough infection rates peak with the increase in number of COVID-19 infections, possibly due to the increased number of vaccinees and the emergence of new SARS-CoV-2 variants. Factors in favor of breakthrough infections included younger ages, male gender, comorbidities, and inactivated vaccines. Study results might support caution regarding relaxation of personal preventive measures in areas with higher or increasing incidence of COVID-19, particularly for the at-risk group even if they are vaccinated.

The study showed that vaccination against COVID-19 still reduces hospital stay and mortality rates, though it does not reduce disease severity much. In order to improve COVID-19 vaccine effectiveness and reduce breakthrough infections, monitoring vaccine effectiveness and breakthrough rate is crucial. Further research is needed to better describe the at-risk group for breakthrough infection.

## Data Availability

The datasets generated and/or analyzed during the current study are not publicly available to protect study subjects’ privacy but are available from the corresponding author (OD) upon reasonable request.

## List of Abbreviations

VBT: Vaccination breakthrough infection
RCT: Randomized Controlled Trial
SARI: Severe Acute Respiratory Illness
RT-PCR: Real-Time polymerase chain reaction
ACE: Angiotensin-Converting Enzyme
ICU: Intensive care unit
